# High-pressure crystallography of periodic and aperiodic crystals

**DOI:** 10.1107/S2052252514025482

**Published:** 2015-01-26

**Authors:** Clivia Hejny, Vasily S. Minkov

**Affiliations:** a Mineralogy and Petrography, University of Innsbruck, Innrain 52, A-6020 Innsbruck, Austria; b Institute of Solid State Chemistry and Mechanochemistry, Siberian Branch of the Russian Academy of Sciences, 18 Kutateladze Street, Novosibirsk 630128, Russian Federation; c Novosibirsk State University, 2 Pirogov Street, Novosibirsk 630090, Russian Federation

**Keywords:** high-pressure crystallography, periodic crystals, aperiodic crystals, incommensurate modulation

## Abstract

More than five decades have passed since the first single-crystal X-ray diffraction experiments at high pressure were performed. These studies were applied historically to geochemical processes occurring in the Earth and other planets, but high-pressure crystallography has spread across different fields of science including chemistry, physics, biology, materials science and pharmacy. With each passing year, high-pressure studies have become more precise and comprehensive because of the development of instrumentation and software, and the systems investigated have also become more complicated. Starting with crystals of simple minerals and inorganic compounds, the interests of researchers have shifted to complicated metal–organic frameworks, aperiodic crystals and quasicrystals, molecular crystals, and even proteins and viruses. Inspired by contributions to the microsymposium ‘High-Pressure Crystallography of Periodic and Aperiodic Crystals’ presented at the 23rd IUCr Congress and General Assembly, the authors have tried to summarize certain recent results of single-crystal studies of molecular and aperiodic structures under high pressure. While the selected contributions do not cover the whole spectrum of high-pressure research, they demonstrate the broad diversity of novel and fascinating results and may awaken the reader’s interest in this topic.

## Abstract 2

This article discusses the high-pressure behaviour of molecular crystal structures, energetic materials, phases relevant to the Earth’s interior, materials with a pressure-induced expansion in one or two directions, dealing with high-pressure data from crystals with twinning and pseudosymmetry, pressure-induced phase transitions including an incommensurate phase, and technical developments.

## 1. Introduction   

At the microsymposium ‘High-Pressure Crystallography of Periodic and Aperiodic Crystals’ of the 2014 IUCr Congress and General Assembly in Montreal, Canada, contributions were presented on the high-pressure behaviour of molecular crystal structures (Dziubek *et al.*, 2014 ▶; Ivanenko *et al.*, 2014 ▶; Kapustin *et al.*, 2014*a* ▶; McGregor *et al.*, 2014 ▶; Olejniczak & Katrusiak, 2014 ▶; Saouane *et al.*, 2014 ▶), energetic materials (Pulham *et al.*, 2014 ▶), phases relevant to the Earth’s interior (Boffa Ballaran *et al.*, 2014 ▶; Miletich *et al.*, 2014 ▶; Pippinger *et al.*, 2014 ▶), materials with a pressure-induced expansion in one or two directions (Cairns & Goodwin, 2014 ▶; Adamson *et al.*, 2014 ▶), dealing with high-pressure data from crystals with twinning and pseudosymmetry (Friese & Grzechnik, 2014*a* ▶, 2014*b* ▶), pressure-induced phase transitions including an incommensurate phase (Bykov *et al.*, 2014 ▶), and technical developments (Munaka *et al.*, 2014 ▶). Although the presented contributions do not cover the whole spectrum of research, they do demonstrate the broad diversity covered by the title and have inspired the chairs of the microsymposium to summarize selected recent results of high-pressure single-crystal studies of molecular and aperiodic structures.

It is now a century since the discovery of X-ray diffraction by Max von Laue and his collaborators (Friedrich *et al.*, 1912 ▶) and the elucidation of the structure of NaCl (Bragg, 1913 ▶), both of which have been celebrated through the International Year of Crystallography (IYCr2014), as declared by the United Nations Educational, Scientific and Cultural Organization (UNESCO) and the International Union of Crystallography (IUCr). Today, X-ray diffraction is the leading technique for studying the structure of matter at the atomic and molecular level. Experimental high-pressure science benefitted from a major breakthrough due to the pioneering work of Percy W. Bridgman (Bridgman, 1931 ▶; McMillan, 2005 ▶), who was awarded the Nobel Prize for his work in 1946, but the beginning of single-crystal X-ray diffraction experiments at high pressure arose from the invention, by Jamieson *et al.* (1959 ▶) and independently by Weir *et al.* (1959 ▶), of the diamond-anvil cell (DAC), a miniature hand-held device for the generation of high pressure that permits simultaneous pressure generation and *in situ* studies of the pressurized material (Bassett, 2009 ▶). For detailed structural studies up to about 15 GPa, single-crystal diffraction was used in the 1970s and 1980s, and is still in use in laboratory-based single-crystal diffractometers (Hazen & Downs, 2000 ▶; McMahon *et al.*, 2013 ▶), whereas angle-dispersive powder diffraction at much higher pressure has became widespread at synchrotron facilities since the 1990s and has been the method of choice for studying twinned crystals or first-order phase transitions of a reconstructive nature, because the latter are usually associated with the crystal not surviving the transition. However, single-crystal diffraction is an absolute necessity for unambiguously solving complicated crystal structures measured *in situ* under high-pressure conditions, and single-crystal studies in the Mbar range have become routine at synchrotron facilities (McMahon *et al.*, 2013 ▶; Merlini & Hanfland, 2013 ▶).

The following major technical developments in the last 20 years have led to a very large increase in the number of structural high-pressure studies in all areas of research. Firstly, the quality of radiation used for diffraction has improved: the advent of third-generation synchrotrons has brought X-ray sources of high brilliance, a wide range of available energies and smaller spot sizes. For laboratory-based diffraction, rotating anodes and microfocus optics for X-ray sources have enabled increases in intensity and resolution, and reductions in the signal-to-noise ratio and exposure times. Secondly, the use of two-dimensional detectors, whether image-plate detectors with both high sensitivity and high dynamic range, CCD detectors with short read-out times or the latest development of solid-state detectors, has greatly improved the quality of the data. Finally, ongoing developments in the design of the DAC have resulted in larger opening angles and less contamination of the sample signal by components of the DAC, thereby providing more complete data sets. To achieve such pressures successfully without diamond failure, the DAC has to be prepared very carefully (Miletich *et al.*, 2000 ▶). In parallel with the technical developments, the design of high-pressure experiments and data-collection strategies (McMahon *et al.*, 2013 ▶; Budzianowski & Katrusiak, 2004 ▶) have been improved and new protocols for data analysis have been developed.

Several constituent components of the DAC, *i.e.* the cell body, the gasket, the backing plates and the diamonds, can interfere or absorb with the primary and diffracted X-ray beams and lead to falsification of the scattered sample intensities (Friese *et al.*, 2013 ▶; Loveday *et al.*, 1990 ▶; Angel, 2004 ▶). Each of the components of the DAC imposes certain problems, so that the measured intensities of sample Bragg reflections have to be corrected, and non-correctable outliers have to be identified and excluded from the set of refined reflections.

Firstly, there is scattering from the gasket (and also from the backing plate, if present). This produces powder rings that overlap with the single-crystal diffraction pattern of the sample, and this scattering must be taken into account. Under certain circumstances this can be avoided experimentally: the combination of a large gasket hole and a small focused primary beam, which are available with state-of-the-art fine-focused X-ray sources or at synchrotron facilities, can remove gasket powder rings, and the use of conically shaped diamonds on a tungsten carbide seat with a sufficiently large opening angle can remove the need for backing plates and their scattering contribution (Boehler & Hantsetters, 2004 ▶; Moggach *et al.*, 2008 ▶).

The diamonds can also cause several effects. Bragg reflections from the diamonds, and also λ/2 reflections from the high-quality and relatively large diamond crystals, overlap the sample reflections and not only misrepresent the integrated intensity of the overlapping sample reflection but also cause incorrect peak-shape parameters from the sample, and this may influence the integrated intensities of all other sample reflections. Furthermore, X-rays scattered by the source-facing diamond can weaken the primary beam so much that sample reflections measured under this condition are not correctable and have to be excluded (Loveday *et al.*, 1990 ▶).

Finally, there are two very different shadowing effects. For large angular values between the cell axis and either the incoming or the scattered X-ray beam, the gasket can shadow part of the beam, especially if the single crystal is so large as to almost touch the gasket wall. If, however, the primary beam is smaller than the gasket hole and also smaller than the sample crystal, the irradiated volume of the sample will become an issue, especially for large and strongly absorbing sample crystals (McMahon *et al.*, 2013 ▶). Another and much stronger shadowing effect is caused by the cell body. With diffraction settings approaching the edge of the angular opening part of the DAC, the two-dimensional detector will be shaded by the DAC and not be irradiated with scattering from the sample inside the DAC. Errors in the calculation of the DAC shadow on the detector might cause the integration of sample reflections at positions that are actually shaded by the DAC. This will cause the respective reflections to be classified as un­observed or, in the case of redundant data sets, the integrated reflection intensities will be averaged from shaded and non-shaded reflections to give wrong values. Both of these shadowing effects have been corrected for successfully.

In order to take into account all of the above-mentioned corrections, a number of programs have been written to aid data analysis of single-crystal data sets measured in a DAC (Friese *et al.*, 2013 ▶; Dera *et al.*, 2013 ▶; Angel & Gonzalez-Platas, 2013 ▶; Petříček *et al.*, 2014 ▶).

Advances in DAC technology have also been made (Gotou *et al.*, 2011 ▶; Boehler & Hantsetters, 2004 ▶; Moggach *et al.*, 2008 ▶). The most promising developments are undoubtedly in the field of combined high-pressure and high-temperature (HP–HT) devices (Dubrovinsky *et al.*, 2010 ▶; Angel *et al.*, 2000 ▶; Boffa Ballaran *et al.*, 2013 ▶) and combined high-pressure and low-temperature (HP–LT) devices (Giriat *et al.*, 2010 ▶; Graf *et al.*, 2011 ▶; Tse *et al.*, 2012 ▶; Li *et al.*, 2012 ▶). The ability to study matter combining these two parameters simultaneously opens the door to a wide range of possibilities. To date, such HT–HP and HP–LT experiments are mainly restricted to synchrotron sources because of the experimental setup, which is larger than for a simple DAC alone and includes numerous flexible cables, so that the mounting of the whole assembly on a laboratory-based single-crystal diffractometer is a very demanding task. For HP–HT experiments, certain high-temperature DACs are available commercially and a wide range of individual designs have been published. These are based on resistive heating applied either internally or externally, in the latter case the whole DAC being heated (Dubrovinskaia & Dubrovinsky, 2003 ▶). Examples of internal resistive heating are a ring-shaped electrical heater that is placed around the gasket within the DAC cell body (Ballaran *et al.*, 2014 ▶), a metallic heating strip on which the sample is placed and then heated, or the use of the gasket itself as a heater (Miletich *et al.*, 2009 ▶). In this way, temperatures of 1273 K have been reached. Other considerations include the development of pressure within the DAC upon temperature change, on account of the different thermal expansivities of the individual components of the DAC. Once temperature equilibrium is reached, the pressure and temperature conditions are very stable, and they are sufficiently stable for the duration of the experimental measurements. A very important issue is the topic of temperature gradient and measuring the temperature of the pressurized sample. Ideally, the temperature sensor must be placed at the position of the sample, which is not possible in reality. The closest approximation to this is to position a *P*–*T* sensor, which can measure both pressure (*P*) abd temperature (*T*) simultaneously, within the sample chamber. Such a *P*–*T* sensor can be any material of known *P*–*T* behaviour and sufficient stability to pressure, temperature and chemical attack, *e.g.* tungsten (Dubrovinsky *et al.*, 2010 ▶). Another option is the use of a thermocouple to measure the temperature. However, its position within the sample chamber or at the back of the diamond has to be considered with great care and attention (Miletich *et al.*, 2009 ▶). A totally different approach to heating the sample in the DAC up to 5000 K is *via* an externally situated laser (Boffa Ballaran *et al.*, 2013 ▶; Dubrovinsky *et al.*, 2009 ▶, 2010 ▶), which has only recently been applied for single-crystalline samples, although it is part of the routine equipment at synchrotron facilities.

It is thus not surprising that the last two decades have brought extraordinary discoveries, with novel and complex new crystal structures and structure types, in the field of experimental high-pressure physics, organic and inorganic chemistry, materials science and earth science, and which are often accompanied by *ab initio* calculations and predictions of structures stable at even higher pressure. The significance of crystallographic studies at high pressure is hard to overestimate: in 2013 and 2014, three special issues devoted to high-pressure and non-ambient crystallography were published in the leading crystallographic and high-pressure journals *Acta Crystallographica*, *Zeitschrift für Kristallographie* and *High Pressure Research*, besides reviews and numerous papers describing original results.

## 2. Twinning and pseudosymmetry at high pressure   

Because of the fact that pressure-induced phase transitions are often accompanied by twinning, there is a requirement to deal with this problem when it arises. The increasing number of published structural refinements performed from high-pressure data sets of twinned crystal structures shows that it is now possible to tackle this problem. A review of available data on pressure-induced twinning (Friese & Grzechnik, 2014 ▶) made the remarkable observation that, for pressure-induced twinning accompanying first-order transitions, the twinning operator is related to the loss of rotational symmetry elements of the higher-symmetry polymorph, although the high- and low-pressure phases are not in a group–subgroup relationship. Moreover, the analysis of pseudosymmetry suggests that the pressure-induced change in the observed pseudosymmetry can be used to predict phase transitions to either higher- or lower-symmetry structures and, in due course, may even be able to predict the stability of the phase under high pressure.

## 3. Pressure-induced phase transitions in energetic materials   

In the search for new energetic materials with enhanced safety properties, such as stability against changes in temperature and humidity, chemical stability, stability against electrical charges and shock resistivity, while simultaneously performing well against the detonation stimulus, simple organic molecules have recently become the focus of attention, *e.g.* 2,4-di­nitro­anisole (DNAN), 1,1-diamino-2,2-dinitro­ethene (DADNE or FOX-7), 1,3,5-trinitro­hexa­hydro-1,3,5-triazine (RDX), cyclo­tetra­methylene (HMX), hexa­nitro­hexa­aza­iso­wurtzitane (CL-20) and 3-nitro-1,2,4-triazol-5-one (NTO) (Pulham *et al.*, 2014 ▶; Coster *et al.*, 2014 ▶; Lloyd *et al.*, 2014 ▶; Hunter *et al.*, 2013 ▶; Millar *et al.*, 2012 ▶; Pulham *et al.*, 2010 ▶; Millar, Marshall *et al.*, 2010 ▶; Millar, Maynard-Casely *et al.*, 2010 ▶; Millar, Oswald *et al.*, 2010 ▶; Oswald *et al.*, 2010 ▶; Davidson *et al.*, 2008 ▶). All of these mol­ecules have the common feature of oxidizing nitro groups in close proximity to an oxidizable hydrocarbon skeleton.

From another point of view, high-pressure crystallographic studies of energetic materials, such as explosives and propellants, provide significant information about processes occurring in the crystalline material during explosion. The operating conditions when a shock wave passes through the explosive can reach very high values of pressure and temperature, of the order of 50 GPa and 5000 K. Undoubtedly, such extreme conditions could induce structural changes or even initiate chemical reactions in the solid. In this respect, *in situ* high-pressure diffraction and spectroscopic studies of such mat­erials provide valuable information about their crystal structures and properties. Results obtained in static compression may then be extrapolated to dynamic shockwave experiments. These empirical measurements give significant input information for experimental computational methods, which are necessary to probe the reaction mechanisms and rates during the high-energy process of explosive decomposition.

The studies of the above-mentioned compounds show a rich pressure- and temperature-dependent polymorphism, with some phases being less sensitive to shock than others, which is of great importance for practical applications. Other important features for practical consideration are the density, crystal morphology and solid–solid phase transitions. These last have been observed for DNAN-based formulations during heat cycling and have produced an irreversible volume increase of up to 15%. In order to understand fully the characteristics of molecular structures used as explosives and to discover new forms of energetic materials with desired properties, such as the above-mentioned reduced sensitivity to accidental initiation, the polymorphs and their character, and the pressure and temperature conditions of their respective transitions, have been studied by X-ray and neutron diffraction of powdered and single-crystalline samples, as well as by computational high-pressure studies.

## 4. Designing next-generation negative compressibility materials   

The volume reduction of matter under the application of hydrostatic pressure is usually achieved by a reduction in the linear dimensions. This is characterized by the linear isothermal compressibility *K*_l_. However, few materials exist which are known to expand in one or even two directions, hence having a negative value for *K*_l_. These materials are called negative linear compressibility materials (NLC) if the expansion occurs in one direction, and negative area compressibility (NAC) materials if the expansion is in two directions (Cairns & Goodwin, 2014 ▶; Cairns *et al.*, 2014 ▶). Simultaneous with the linear expansion, NLC and NAC materials contract in the direction perpendicular to the expansion, because the intrinsic volume compressibility has to be positive. It can therefore be concluded that, in the search for new materials with high negative linear compressibility, it is a necessity that the material is anisotropic and has at least one direction with a very large compressibility. Materials with a giant NLC behaviour have *K*_l_ < −30 TPa^−1^ and the phase is stable over a pressure range of at least 1 GPa, *e.g.* *K*_l_ = −42 (5) TPa^−1^ for Zn[Au(CN)_2_]_2_, which is stable from ambient conditions up to 1.8 GPa.

Inspection of the structural features and their behaviour under pressure shows that this giant NLC response can be understood due to the concurrence of two effects. Firstly, in the β-quartz-like framework structure of Zn[Au(CN)_2_]_2_, honeycomb-like channels parallel to the *c* axis simultaneously elongate along the *c* direction and become narrower within the (001) crystal plane. This is a geometric effect, similar to the so-called wine-rack mechanism found in other NLC materials. Secondly, Au⋯Au interactions in the form of helices perpendicular to the *c* axis can act as springs. For a spring, the compressibility is larger than for the individual interaction itself, and indeed the repeat length of the helix decreases much more strongly than the average Au⋯Au distance. Interestingly, this is a purely pressure-driven mechanism, in contrast with the thermally activated changes in the Au⋯Au distances. This opens the field for a wide range of applications, *e.g.* highly sensitive optical components in interferometric pressure sensors. Identification of the structural motifs and understanding of the underlying mechanism responsible for NLC effects allow the search for new materials with the most extreme NLC effects, as has been demonstrated for Zn[Au(CN)_2_]_2_ and also for the even rarer materials with negative area compressibility, *e.g.* Ag-tricyanomethanide (Hodgson *et al.*, 2013 ▶).

## 5. High-pressure crystallography of molecular crystals   

High-pressure X-ray diffraction studies of molecular crystals differ in several respects from those of crystals of inorganic compounds. On account of the weak scattering factors of atoms of the lighter elements (*e.g.* H, C, N, O), the low symmetries and densities of molecular crystals, their large unit-cell dimensions and the high thermal vibrations of their atoms, the reflections diffracted by molecular crystals are often more difficult to measure precisely or even to detect above the background of the radiation scattered from components of the high-pressure cell, especially at higher 2θ angles. Nevertheless, structural studies of the crystals of organic compounds by diffraction techniques at high pressure began immediately after the DAC had been invented. The first studies go back to the end of 1960s, when Fourme (1968 ▶) published the determination of the crystal structure of chloroform and Piermarini *et al.* (1969 ▶) solved the high-pressure phase of benzene. After almost five decades of continuous development in instrumentation and software, high-pressure crystallographic studies of molecular crystals are now becoming more a matter of routine. At the same time, the systems under study have become more complicated: small organic molecules have been replaced by molecules consisting of a large number of atoms with large unit-cell dimensions, low symmetry and several crystallographically independent formula units, as well as macromolecules such as proteins and peptides, or even cellular organelles. The objectives and goals of such studies have been expanded and deepened. Nowadays, the solution of a crystal structure from single-crystal data collected using a DAC is quite a common procedure. Recent papers describe high-pressure-induced conformational and structural changes and phase transitions in crystal structures. Current high-pressure studies provide valuable information on the subtle changes and processes occurring: distortion of intra- and intermolecular interactions and hydrogen bonds, high-pressure crystallization processes, formation of host–guest compounds and solvates, triggering of new chemical reactions, polymerization, amorphization *etc*. High pressure is also used as a powerful tool for studying polymorphism, which is very important for molecular crystals since their properties depend on crystal structure. In addition to valuable fundamental knowledge, high-pressure studies of organic crystals are of significant practical importance, since molecular crystals are used as drugs and as ferroelectric, piezoelectric, pyroelectric and nonlinear optical materials (Boldyreva, 2008 ▶, 2014 ▶).

Among the many papers devoted to high-pressure X-ray diffraction studies of molecular crystals, one can distinguish three general experimental techniques based on the type of system being investigated. The first and most popular approach can be applied to materials which are already crystalline under ambient conditions. Studies of molecular crystals at variable pressure provide an insight into the nature and dynamics of intra- and intermolecular interactions, particularly hydrogen bonds, the distortion of crystal packing, the switching of molecular conformations, proton migration between donor and acceptor groups, reconstructive phase transitions, the formation of high-pressure phases *etc*. The second technique considers isochoric and isothermal high-pressure crystallization of organic compounds which are liquid at ambient temperature and pressure. Increasing the pressure inside a DAC leads to crystallization or freezing of the liquid sample. Alongside low temperature, high pressure can assist the crystallization of an initially liquid compound, and on many occasions the low-temperature and high-pressure phases may be different. Nevertheless, by varying the crystallization conditions (pressure and temperature) one can obtain different high-pressure polymorphs. The third technique is probably the least common and concerns high-pressure crystallization of solute organic molecules from solution, often leading to the formation of hydrates and solvates. In general, compression, similar to cooling, reduces the solubility of a substance. Such an approach allows one to study the formation of new high-pressure polymorphs and solvates which cannot be obtained on crystallization under ambient conditions, nor even after phase transitions induced by increasing pressure in the starting phase. Sometimes, high-pressure polymorphs crystallized by this technique are stable under ambient conditions for a long time after opening the high-pressure cell. This has applications in the production of new polymorphs or solvates of pharmaceutical compounds.

The group of Professor Katrusiak at Poznan University, Poland, is one of the pioneers of the high-pressure X-ray diffraction technique, and they have been systematically investigating organic crystals at high pressure since the end of the 1980s. Starting with high-pressure crystallographic studies of crystals of squaric acid published in 1986 (Katrusiak & Nelmes, 1986 ▶) and 1,3-cyclohexanedione published in 1990 (Katrusiak, 1990 ▶), many structures of organic crystals at high pressure have been determined and reported by this team. Taking into account the 1990s, the ‘dark times’ for high-pressure crystallography, when there were no modern high-sensitivity area detectors, bright X-ray sources, high-performance computers or dedicated data-processing software, these pioneering works of high-pressure diffraction structural studies of molecular crystals were very difficult and time-consuming. Professor Katrusiak’s group have developed and successfully applied new approaches and methodologies of high-pressure X-ray diffraction crystallography (Katrusiak, 1999*a* ▶,*b* ▶; Dera & Katrusiak, 1999 ▶; Dera & Katrusiak, 2001 ▶; Dziubek & Katrusiak, 2002 ▶; Katrusiak, 2004*a* ▶,*b* ▶; Katrusiak, 2008 ▶). During the last decade, the group has focused on the high-pressure crystallization in DACs of organic compounds which are liquid at ambient temperature and atmospheric pressure. The liquid organic substance fills the high-pressure chamber of a DAC and is compressed to the particular pressure where the transition from liquid to solid occurs. Since it is hard to increase pressure gradually, the compound usually crystallizes as a polycrystalline mass. In order to obtain a single crystal, one needs to reliquefy the polycrystalline material by varying the temperature or volume/pressure, so these methods are called isothermal and isochoric high-pressure crystallization. The easier and therefore more widespread method is to heat the high-pressure chamber until only one small crystal is left, and then allow the cell to cool slowly to ambient temperature to induce growth of this small crystal. The isothermal method is more complicated since one needs to decrease the pressure (by increasing the volume) very accurately in order to reliquefy all of the small crystallites except one, and then gradually increase the pressure (by decreasing the volume) to allow the small crystal to grow. In this respect, gas–membrane high-pressure cells are very helpful for the purposes of the isochoric method. Both approaches are very challenging, and quite often it is hard to isolate only one crystal from a polycrystalline sample or to eliminate the formation of other crystals when the desired one is growing.

At the microsymposium the group was represented by Dr Olejniczak and Dr Dziubek. The research of Dr Dziubek is focused on the investigation of halogen⋯halogen intermolecular interactions in the structures of the molecular crystals of several halogeno­alkanes (Podsiadło *et al.*, 2005 ▶, 2006 ▶; Dziubek & Katrusiak, 2008 ▶; Dziubek *et al.*, 2009 ▶) and halogeno­arenes (Bujak *et al.*, 2007 ▶; Dziubek *et al.*, 2014 ▶). For instance, such interactions can be considered as the main cohesive forces responsible for the molecular arrangement in such systems. His studies are also dedicated to the understanding of the role of specific and weak C—H⋯π and C—H⋯O hydrogen bonds in pressure-frozen phases of ethynyl­benzene (Dziubek *et al.*, 2007 ▶) and tetra­hydro­furan (Dziubek *et al.*, 2010 ▶). Particularly, he showed that, in the case of isomers of dibromo- and dichloro­benzenes, their melting points correspond not only to Carnelley’s rule concerning molecular symmetry, but also in terms of the number of intermolecular halogen⋯halogen interactions in the crystal structure. In other words, the higher the number of such contacts per molecule in the structure, the higher the melting point of the crystal (Bujak *et al.*, 2007 ▶; Dziubek & Katrusiak, 2014*a* ▶). A comparative study of the low-temperature and high-pressure phases of trihalo­methanes (H atoms are substituted by Cl and Br) has shown that molecular symmetry controls molecular aggregation in the crystalline state, consistent with the crystal site symmetry and the balance of electrostatic matching and dispersion forces between molecules (Dziubek & Katrusiak, 2008 ▶; Dziubek *et al.*, 2009 ▶). Study of the pressure-freezing of ethynyl­benzene made it possible to resolve  C—H⋯π(arene) and cooperative  C—H⋯π(C C) inter­actions (Dziubek *et al.*, 2007 ▶). The current results of Dr Dziubek are related to temperature-variable high-pressure volumetric studies of diethylene glycol. Volumetric measurements in a piston and cylinder press are of special interest and have been demonstrated to be a complementary method to diffraction experiments in a DAC. Such measurements provide precise data on volume compression for liquids and solids, in particular volume changes during pressure-freezing of liquids and solid-to-solid phase transitions (Dziubek & Katrusiak, 2014*b* ▶). Applying temperature as a second parameter, this relatively simple and rapid method becomes very efficient for exploring the phase diagrams of compounds and volume reduction at phase transitions.

Dr Olejniczak also has great experience in the high-pressure crystallization of liquids or solutions of solid compounds, such as penta­fluoro­pyridine (Olejniczak *et al.*, 2008 ▶), aceto­nitrile (Olejniczak & Katrusiak, 2008 ▶), 2-bromo-2-chloro-1,1,1-tri­fluoro­ethane (Olejniczak, Katrusiak *et al.*, 2009 ▶), dihalo­perfluoro­ethanes (Olejniczak, Katrusiak & Vij, 2009*a* ▶,*b* ▶), urea (Olejniczak, Katrusiak *et al.*, 2009 ▶; Olejniczak, Katrusiak & Vij, 2009*a* ▶,*b* ▶; Olejniczak, Ostrowska & Katrusiak, 2009 ▶), thiourea hydrates (Tomkowiak *et al.*, 2013 ▶) and chloro­aceto­nitriles (Olejniczak & Katrusiak, 2011*a* ▶). The current interest of Dr Olejniczak is focused on the structures and properties of 1,4-diazabicyclo[2.2.2]octane (dabco) mono­salts at high pressure. The system consists of a number of compounds with the general formula dabcoH*X*, where *X* is Br^−^, I^−^, ClO_4_^−^, BF_4_^−^ or ReO_3_^−^. They possess dielectric and anisotropic relaxor properties and are very promising as new ecologically safe materials (Olejniczak *et al.*, 2010 ▶, 2013 ▶; Andrzejewski *et al.*, 2011 ▶, 2012 ▶; Anioła *et al.*, 2014 ▶; Olejniczak & Katrusiak, 2010 ▶, 2011*b* ▶; Nowicki *et al.*, 2012 ▶). Dr Olejniczak has shown that dabcoHI undergoes a large number of transformations at elevated pressure and temperature, but that the analogous dabcoHBr exists in only three forms. An exceptionally rich phase diagram of ten phases was also revealed for dabcoHClO4. At normal conditions in dabco monosalts, the dabcoH^+^ cations are bonded by NH^+^⋯N hydrogen bonds into linear chains, but pressure can considerably modify that pattern, and weak hydrogen bonds are relevant for the formation of new polymorphs at high pressure. In all dabcoHI polymorphs, the NH^+^⋯N bonded chains of dabcoH^+^ cations are retained. However, the chains are linear at 0.1 MPa and high pressure induces modulation of the chain with gradually increased periodicity. The polymorphic structures of dabco salts differ mainly in the arrangement of the chains and corresponding anions, in the dabco conformation, and in the location of the protons.

High-pressure crystallization of molecular crystals from their saturated solutions by careful tuning of temperature and pressure in a DAC is well developed and commonly applied by the group of Dr Fabbiani at Göttingen University, Germany. After only a decade, since the first new crystal structure of a methanol solvate of paracetamol was published in 2003 (Fabbiani *et al.*, 2003 ▶), a series of crystals have been crystallized in a DAC by means of this *in situ* technique and their structures have been determined, for example ciprofloxacin sodium salts (Fabbiani *et al.*, 2009 ▶), gabapentin heptahydrate (Fabbiani, Levendis *et al.*, 2010 ▶), piracetam (Fabbiani, Allan, Parsons & Pulham, 2005 ▶) and its hydrate (Fabbiani *et al.*, 2007 ▶), acetamide and parabanic acid hydrate (Fabbiani, Allan, Marshall *et al.*, 2005 ▶), vitamin B hydrate (Fabbiani, Buth *et al.*, 2010 ▶), and naphthalene, phenanthrene and pyrene (Fabbiani *et al.*, 2006 ▶). Most of these compounds are used as active pharmaceutical ingredients in drugs, and therefore the study of their crystal structures and properties is of great practical importance. At the same time, there is interest in developing understanding of the thermodynamic and kinetic factors of the formation of crystal structures from fluid phases, as well as their response to varying temperature and pressure, particularly phase transitions. One of the main problems in the pharmaceutical industry is to find the required experimental conditions in order to ensure the stable and reproducible crystallization of the desired polymorphic modification of a particular compound. Conventional polymorph screening is limited by recrystallization of the substance from a number of solvents under various conditions (temperature, pH, moisture, agitation, sonication *etc*). High-pressure crystallization is a complementary approach to explore polymorphism in mol­ecular crystals that has been successfully demonstrated (Oswald *et al.*, 2009 ▶). Moreover, after decompression and opening of a DAC, some crystallized high-pressure phases do not transform to the ambient-pressure phase and are preserved at room temperature and atmospheric pressure for a long time, but this is still a rather uncommon phenomenon. In addition, crystalline high-pressure phases produced by this method can be used for seeding and growth of the corresponding phase from solution under ambient conditions. It was recently shown that *in situ* high-pressure crystallization of γ-amino butyric acid from a variety of aqueous solutions reproducibly yields its monohydrate in a pressure range of 0.4–0.8 GPa, whereas crystallization under ambient conditions invariably leads to the anhydrous monoclinic form of the compound (Fabbiani *et al.*, 2014 ▶). Although the structure of γ-amino butyric acid monohydrate is thermodynamically more favourable at high pressure over two anhydrous forms, adding seeds of this phase recovered at ambient pressure to a saturated aqueous solution of this carboxylic acid provokes the hydrate crystals to grow. This study shows a very powerful tool to produce crystalline forms which are unobservable or elusive under ambient conditions, using seeds of high-pressure phases obtained by *in situ* crystallization. Nonetheless, the technique of *in situ* high-pressure crystallization of molecular crystals from solution is still an emerging field and its huge potential as a tool for controlling polymorphism and the formation of solvates has not yet been fully comprehended and realised.

The current research by the group of Dr Fabbiani concerns the *in situ* high-pressure crystallization of some very interesting and novel molecular inclusion complexes based on β-cyclodextrin, one of the most commonly used compounds in host–guest supramolecular chemistry. Having a hydrophilic outer surface and a non-polar inner cavity, cyclodextrin is often used to encapsulate suitable small molecules. Since cyclodextrin is very soluble in water, the solubility of such host–guest complexes is much higher than for the pure intercalated compound, and this method is commonly used in the pharmaceutical industry. The idea of studying the crystallization of host–guest complexes at high pressure appeared only very recently. At the end of 2012, the group published a new crystal structure of the novel hydrate of α-cyclodextrin crystallized at 0.65 GPa (Granero-García *et al.*, 2012 ▶). At the beginning of 2014, they published the crystal structure of the first simple inclusion complex of β-cyclo­dextrin and dimethyl­formamide (Granero-García & Fabbiani, 2014 ▶). The presentation at the microsymposium is a logical continuation of this work. The authors have made the task more difficult and chosen the active pharmaceutical ingredient, paracetamol, as a guest molecule for the β-cyclodextrin host. They described compression studies of this inclusion complex in different crystallization media at pressures up to 1 GPa, as well as characterization of the new high-pressure phase by different techniques (polarized optical microscopy, Raman spectroscopy, and single-crystal X-ray diffraction using both home and synchrotron sources). It is worth noting that such experiments are the next level in crystal engineering, since there are many more different fragments to self-assemble in the investigated system, and one has to control not only the crystallization of the required polymorph, but also of a supramolecular multicomponent crystal *versus* several separate monocomponent phases. By increasing the number of components to two or three, one can modify or even radically rearrange the crystal structure and, in this way, change the physical properties of a solid in a controlled way (Boldyreva, 2014 ▶).

The group of Professor Boldyreva at Novosibirsk State University, Russian Federation, is also one of the pioneers of high-pressure crystallography, focusing on studies of the effect of pressure on solid molecular compounds immersed in hydrostatic liquids. Alongside the high-pressure crystallization of liquids and solutions, such studies are of fundamental significance, increasing understanding of the nature of intra- and intermolecular interactions in crystals, in particular hydrogen bonds, and providing better comprehension of the polymorphism of molecular crystals and the mechanisms of structural rearrangement during phase transitions and solid-state reactions (Boldyreva, 2008 ▶, 2009 ▶, 2014 ▶). The first high-pressure study of molecular crystals was done by this group for paracetamol polymorphs in the early 2000s (Boldyreva *et al.*, 2000 ▶; Boldyreva, Shakhtshneider & Ahsbahs, 2002 ▶). After this first batch of experiments, high-pressure effects on crystal structure and properties were studied for a number of mol­ecular solids, including pharmaceutical substances (Boldyreva, Shakhtshneider, Ahsbahs, Uchtmann *et al.*, 2002 ▶; Boldyreva, Dmitriev & Hancock, 2006 ▶; Seryotkin *et al.*, 2013 ▶; Minkov & Boldyreva, 2013 ▶), amino acids (Boldyreva, Ahsbahs & Weber, 2003 ▶; Boldyreva *et al.*, 2004 ▶; Boldyreva, Ivashevskaya *et al.*, 2005 ▶; Boldyreva, Kolesnik *et al.*, 2005 ▶, 2006 ▶; Boldyreva, Sowa *et al.*, 2006 ▶; Drebushchak *et al.*, 2006 ▶; Minkov, Tumanov *et al.*, 2010 ▶; Tumanov *et al.*, 2008 ▶, 2010 ▶; Tumanov & Boldyreva, 2012 ▶; Zakharov *et al.*, 2012 ▶), salts of carboxylic acids and alkaline metals (Boldyreva, Shakhtshneider, Ahsbahs, Sowa & Uchtmann, 2002 ▶; Boldyreva, Goryainov *et al.*, 2003 ▶; Boldyreva, Kolesnik *et al.*, 2006 ▶; Boldyreva, Sowa *et al.*, 2006 ▶; Boldyreva, Ahsbahs *et al.*, 2006 ▶), and multicomponent crystals or co-crystals (Minkov *et al.*, 2012 ▶; Zakharov & Boldyreva, 2013 ▶; Zakharov *et al.*, 2013 ▶). One of the strengths of the group is the application of several different instrumental techniques, in particular combining diffraction and spectroscopy techniques. X-ray diffraction methods give direct information on the positions of atoms and their atomic displacement parameters, molecular conformations, and intra- and intermolecular interactions and contacts. At the same time, spectroscopic methods have an obvious advantage as far as estimates of the energies of intermolecular interactions, particularly hydrogen bonds, and the study of the dynamics of selected molecular fragments are concerned. Thus, such a combination of several techniques has a synergistic effect, giving a better understanding of the very subtle features of dynamics in the solid state. Such an approach has been demonstrated to be very powerful for the investigation of high-pressure induced effects in several systems (Kolesnik *et al.*, 2005 ▶; Minkov *et al.*, 2008 ▶, 2012 ▶; Minkov, Tumanov *et al.*, 2010 ▶; Minkov, Goryainov *et al.*, 2010 ▶; Zakharov *et al.*, 2012 ▶; Minkov & Boldyreva, 2013 ▶; Kapustin *et al.*, 2014*b* ▶).

Very recent results from the group include a high-pressure study of the relative role of charge-assisted N—H⋯O hydrogen bonds and steric repulsion of methyl groups in a range of *N*-methyl derivatives of glycine. It is well known that infinite head-to-tail chains built by N—H⋯O hydrogen bonds are common structural motifs in crystals of amino acids and coincide with the most rigid direction in the crystal structure on cooling and increasing pressure. However, one should take into account the dual nature of these chains: despite the hydrogen bonds, there are also electrostatic interactions between the zwitterions. Guided by an idea to distinguish the electrostatic contribution from the charge-assisted N—H⋯O hydrogen bonds and to understand their role in crystal structure distortion on increasing pressure, the crystal structures of mono-, di- and trimethyl derivatives of glycine were considered as a case study. Methylation not only excludes the formation of selected hydrogen bonds but also introduces bulky mobile fragments into the structure. The effects of pressure on the systems of the series were compared with respect to distorting and switching hydrogen bonds and inducing reorientation of the methyl fragments. In all the structures studied, increasing pressure results in compression of head-to-tail chains. After a particular degree of structure compression is achieved, the unfavourable short contacts seek to be expelled through a phase transition. If there are no hydrogen bonds in the structure, the zwitterions ‘gradually’ change their orientation within the chain; in a structure with hydrogen bonds, any distortion of the chain is accompanied by changes in the N—H⋯O hydrogen bonds which act as springs, provoking sudden structural changes. In dimethyl­glycine, the sole hydrogen bond is apparently not sufficient to hold the strain imposed on the crystal structure by the increased pressure, and the structure collapses at very low pressure (Kapustin *et al.*, 2014*a* ▶,*b* ▶).

## 6. High-pressure crystallography of aperiodic crystals   

Aperiodic crystal structures have long-range order but lack translational symmetry. They are subdivided into quasi­crystals, incommensurate composite structures (also termed host–guest structures) and incommensurately (IC) modulated structures. Although high-pressure studies of all three kinds of aperiodic crystals are available and will very briefly be reviewed in the following, this research field is just about to take off. The presence of IC modulated crystals has puzzled scientists since the beginning of the last century, when it was noticed that some minerals form crystal faces that cannot be indexed with three integer values, and which also provide diffraction patterns in which the diffraction maxima cannot be indexed based on any of the known lattices with the conventional three Miller indices (Smith, 1903 ▶; Goldschmidt *et al.*, 1931 ▶; Donnay, 1935 ▶), even if twinning is considered. However, the understanding of IC modulated structures and the development of a mathematical tool to describe them, *i.e.* higher-dimensional crystallography or the so-called superspace approach (Janssen *et al.*, 2004 ▶; van Smaalen, 2004 ▶), evolved slowly and the term ‘incommensurate phase’ was not introduced until the early 1970s (DeWolff, 1974 ▶; Janner & Janssen, 1977 ▶). It took several more decades for the development of easy-to-use software with which the routine analysis of modulated crystal structures is now possible, *e.g.* *JANA2006* (Petříček *et al.*, 2014 ▶), which also implements tools for the analysis of high-pressure data sets. Today, the superspace approach is successfully applied to crystal structures with incommensurate and commensurate modulation, whereby the latter can also be refined in terms of a superstructure (Bykov, Bykova *et al.*, 2013 ▶). This eases the comparison of interrelated incommensurate and commensurate structures, but additionally it is also of special interest for the refinement of high-pressure data, because of the reduction in the number of parameters in the refinement process. The 1980s brought the discovery of quasicrystals (Shechtman *et al.*, 1984 ▶), which led to the redefinition of the term ‘crystal’ by means of the presence of essentially sharp Bragg reflections in a diffraction pattern (IUCr, 1992 ▶), rather than the former definition of three-dimensional translational periodicity; this work was awarded the Nobel Prize in chemistry in 2011.

The combination of the two fields, high-pressure science and aperiodic crystallography, demands special expertise for sample preparation, data collection and data processing. Therefore, high-pressure studies of aperiodic materials have been rare until recently as they represent a great challenge. However, the first high-pressure study of an IC modulated structure was performed over 20 years ago (Reithmayer *et al.*, 1993 ▶) on calaverite, AuTe_2_. Under ambient conditions, calaverite has large structural displacements of the Te atoms of about 0.4 Å, which decrease upon increasing pressure up to 2.5 GPa, where a reversible first-order phase transformation takes place.

**Figure 1:**
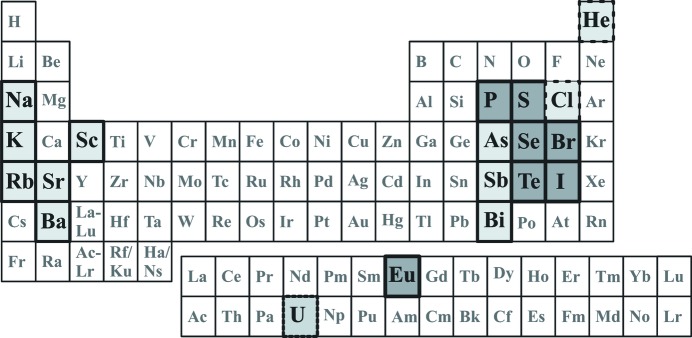
The periodic table of the elements, highlighting elements with an aperiodic structure at high pressure. (i) P, S, Se, Te, Br, I and Eu, with an experimentally determined IC modulated crystal structure, are highlighted in dark grey with a thick border. (ii) He and Cl, with a predicted IC modulated structure, are highlighted in light grey with a long-dashed border. (iii) U, with an IC charge-density wave at low temperature, is highlighted in light grey with a short-dashed border. (iv) Na, K, Rb, Sr, Ba, Sc, As, Sb and Bi, with an IC composite structure, are highlighted in light grey with a thick border.

‘One of the most exciting developments of recent years’, as considered by experts in the field (van Smaalen, 2004 ▶), is the discovery of incommensurate composite and IC modulated crystal structures of the elements (Fig. 1 ▶) at high pressure. The first element for which an incommensurate structure was reported is uranium, with an IC modulated charge-density wave at low temperatures (van Smaalen & George, 1987 ▶). Subsequently, an IC composite structure was found for the high-pressure phase Ba-IV (Nelmes *et al.*, 1999 ▶). The Ba atoms form two different types of interpenetrating substructures, a host tetragonal framework structure and a guest structure formed of linear chains within channels of the host framework. In two directions the host and guest are commensurate, but along the channel and chain direction the ratio formed by the translational period of the host atoms and that of the guest atoms is an irrational number, so the two substructures are incommensurate. Several IC phases with the same host structure but different guest structures have been reported for Ba. Very similar commensurate and incommensurate host–guest structures, with basically the same host structure or distortions of it, have also been found for high-pressure phases of Na, K, Rb, Sr, As, Sb, Bi and Sc (McMahon & Nelmes, 2004 ▶; McMahon & Nelmes, 2006 ▶; McMahon *et al.*, 2006 ▶; Degtyareva, 2010 ▶; Lundegaard *et al.*, 2009 ▶, 2013 ▶).

IC modulated crystal structures have subsequently been found in high-pressure phases of I, Te, S, Se, P, Br and Eu (Takemura *et al.*, 2003 ▶; Hejny & McMahon, 2003 ▶; McMahon *et al.*, 2004 ▶; Hejny *et al.*, 2005 ▶; Duan *et al.*, 2010 ▶; McMahon & Nelmes, 2006 ▶) and have been suggested for He and Cl on the basis of first-principles calculations (Li *et al.*, 2012 ▶) or Monte Carlo simulations (Cazorla *et al.*, 2013 ▶). For iodine, it could be shown that the IC phase is a transitional structure between the molecular low-pressure phase with dimerized I_2_ and the atomic high-pressure phase with a monatomic arrangement of the iodine atoms. The IC modulated structure of iodine contains parts in which the atomic distances resemble those of the high-pressure atomic form. These parts increase in volume with pressure approaching the phase transition to the high-pressure phase. The stability of the IC phases and the pressure dependence of the modulation, especially the modulation wavevector and interatomic distances, have also been studied as a function of pressure and temperature at both high and low temperature (Hejny *et al.*, 2006 ▶; Tse *et al.*, 2012 ▶; Desgreniers *et al.*, 2014 ▶). The latest studies of the complex structures of the dense elemental phases show their instability at low temperature (Tse *et al.*, 2012 ▶; Desgreniers *et al.*, 2014 ▶). Fig. 1 ▶ gives a summary of currently known elements with IC modulated structures at high pressure.

Among the growing number of structural investigations of the high-pressure dependence of IC modulated structures (*e.g.* Merlini *et al.*, 2009 ▶; Hejny *et al.*, 2009 ▶, Ardit *et al.*, 2012 ▶), there is a notable accumulation of IC modulated structures in conjunction with magnetism and superconductivity, *e.g.* RbFe(MoO_4_)_2_ (Kozlenko *et al.*, 2013 ▶), TiSe_2_ (Joe *et al.*, 2014 ▶), Mn_2_GeO_4_ (Honda *et al.*, 2014 ▶) and TiPO_4_ (Bykov, Zhang *et al.*, 2013 ▶; Bykov *et al.*, 2014 ▶ ▶). For TiPO_4_ it was shown that the same IC modulated structure has been observed at high pressure above 6 GPa (Bykov *et al.*, 2014 ▶), after the existence of this structure had been proven at low temperature between 75 and 112 K (Bykov, Bykova *et al.*, 2013 ▶). The lock-in phase transition to a superstructure or commensurately modulated structure at 75 K is comparable with the lock-in transition of the high-pressure phase to a commensurate phase at 7.2 GPa, which is, on further pressure increase, stable up to 45 GPa (Bykov *et al.*, 2014 ▶). Structurally, the modulation strongly influences the Ti atomic positions. This causes a dimerization, which enables a direct exchange interaction between the spins of neighbouring Ti atoms.

The stability of quasicrystals has been examined as a function of temperature, of pressure, and of temperature and pressure (Krauss & Steurer, 2003 ▶; Yamada *et al.*, 2014 ▶). Although quasicrystals are not close-packed structures and hence phase transitions are expected at high pressure, they show remarkably stable behaviour, with the only reported pressure-induced phase transitions being in i-AlLiCu (Krauss & Steurer, 2003 ▶; Krauss *et al.*, 2003 ▶) and in the Zn_6_Sc approximant to a quasicrystal (Yamada *et al.*, 2014 ▶). In no other high-pressure study of quasicrystals has a phase transition been observed in the investigated pressure range of up to 70 GPa. In addition to this remarkable pressure stability, quasicrystals are also stable over cosmic timescales. The first and only natural quasicrystal, icosahedrite Al_63_Cu_24_Fe_13_, was found in the Khatyrka meteorite. It was formed by an impact-induced shock in the early solar system from a melt of Al,Cu-bearing minerals at least 4.5 billion years ago (Hollister *et al.*, 2014 ▶). This naturally occurring quasicrystalline material has also been demonstrated to be stable over a considerable range of pressure and temperature (Stagno *et al.*, 2014 ▶).

## 7. Conclusions   

The latest research in high-pressure crystallography shows very detailed results that could not have been achieved one or two decades ago. Technical and method­ological advances allow the detection of diffraction from even weakly scattering materials and of other weak features, such as satellite reflections. This allows the discussion of the different hydrogen-bond patterns of polymorphs or of the modulation of atomic position, to give just two examples, and hence enables a thorough discussion of structural and property-related implementations.
